# Can we spot fake public comments generated by ChatGPT(-3.5, -4)?: Japanese stylometric analysis expose emulation created by one-shot learning

**DOI:** 10.1371/journal.pone.0299031

**Published:** 2024-03-13

**Authors:** Wataru Zaitsu, Mingzhe Jin, Shunichi Ishihara, Satoru Tsuge, Mitsuyuki Inaba

**Affiliations:** 1 Faculty of Psychology, Mejiro University, Tokyo, Japan; 2 Institute of Interdisciplinary Research, Kyoto University of Advanced Science, Kyoto, Japan; 3 Speech and Language Laboratory, Australian National University, Canberra, Australia; 4 School of Informatics, Daido University, Aichi, Japan; 5 College of Policy Science, Ritsumeikan University, Kyoto, Japan; National Institute of Informatics, JAPAN

## Abstract

Public comments are an important opinion for civic when the government establishes rules. However, recent AI can easily generate large quantities of disinformation, including fake public comments. We attempted to distinguish between human public comments and ChatGPT-generated public comments (including ChatGPT emulated that of humans) using Japanese stylometric analysis. Study 1 conducted multidimensional scaling (MDS) to compare 500 texts of five classes: Human public comments, GPT-3.5 and GPT-4 generated public comments only by presenting the titles of human public comments (i.e., zero-shot learning, GPT_zero_), GPT-3.5 and GPT-4 emulated by presenting sentences of human public comments and instructing to emulate that (i.e., one-shot learning, GPT_one_). The MDS results showed that the Japanese stylometric features of the public comments were completely different from those of the GPT_zero_-generated texts. Moreover, GPT_one_-generated public comments were closer to those of humans than those generated by GPT_zero_. In Study 2, the performance levels of the random forest (RF) classifier for distinguishing three classes (human, GPT_zero_, and GPT_one_ texts). RF classifiers showed the best precision for the human public comments of approximately 90%, and the best precision for the fake public comments generated by GPT (GPT_zero_ and GPT_one_) was 99.5% by focusing on integrated next writing style features: phrase patterns, parts-of-speech (POS) bigram and trigram, and function words. Therefore, the current study concluded that we could discriminate between GPT-generated fake public comments and those written by humans at the present time.

## Introduction

Currently, we are facing an unprecedented crisis caused by artificial intelligence (AI). The proliferation of disinformation such as fake news and images may begin to surround us without our recognition. ChatGPT [1] has played a major role in sparking the beginning. This large language model (LLM), trained and released by OpenAI on November 30, 2022, naturally generates human-like text. Recent chatbots have a generative pretrained transformer (GPT), which dramatically improves the generative performance. These chatbots are convenient and provide various benefits, but it is easy to imagine many kinds of problems, such as manipulating public opinion, writing fake customer reviews, and submitting fabricated academic papers. It has already become possible for anyone to easily generate a large amount of fake public comments for the purpose of making the government create laws and regulations in line with one’s own opinions. To make matters worse, previous studies [2, 3] have verified that almost no people can distinguish between AI-generated and human-written sentences at first glance. Such social problems have already arisen worldwide. Therefore, controlling and understanding generative AI is an urgent issue for humans. The purpose of this study is to try to classify human public comments and ChatGPT-generated fake public comments.

Several researchers have reported the possibility of distinguishing between ChatGPT-generated and human-written texts [4, 5]. Desaire et al. [4] made ChatGPT-3.5 learn human-written academic papers as training data and compared ChatGPT-generated and human-written texts. Zaitsu & Jin [5] also gave instructions against ChatGPT to generate texts by presenting the titles of Japanese scientific academic papers. The results of these studies were distinguishable with nearly 100% accuracy. However, several studies for distinguishing AI-generated and human-written sentences exist. Therefore, it is necessary to conduct research that targets various genres. Brown et al. [6] proposed a learning method without changing the parameters of GPT-3, such as fine-tuning: zero-shot, one-shot, and few-shot learning. One-shot or few-shot learning attempts to obtain an answer by providing prompts with any additional information, whereas zero-shot learning only provides instructions without other information. A question arises here: when we present human-written text as a sample against AI and instruct them to emulate the contents and writing styles of the example, can we distinguish between AI-emulated and human-written texts? In this study, we compared ChatGPT-generated fake public comments with and without emulation (i.e., zero-shot or one-shot learning) to human-written true public comments. This study will make a great contribution to the solutions of problems and risks facing modern society, especially manipulating public opinions using fake public comments generated by ChatGPT.

Public comments (or public consultations) are important civic opinions in establishing rules and orders, such as laws and regulations, and differ from academic papers in two ways: (1) Higher degree of freedom in writing styles because public comments have fewer constraints. (2) Public comments (a few hundred characters) have fewer word counts than academic papers (over thousands of characters). It is expected that the higher the degree of freedom for writing, the easier it is to discriminate the texts of both AI and humans because the features of writing styles are easily expressed. On the other hand, the fewer the word counts, the more difficult the discrimination because the amount of information available for distinguishing decreases.

In study 1, we prepared sample through following methods: (1) “HM” texts (100 samples): human public comments published by Japanese national administrative agencies, (2) “GPT3.5_zero_” or “GPT4_zero_” texts (every 100 samples): ChatGPT (GPT-3.5 and -4)-generated texts with only presenting the title of public comments (zero-shot learning), (3) “GPT3.5_one_” or “GPT4_one_” texts (each 100 sample): We instructed ChatGPT (GPT-3.5 and -4) to emulate the contents and writing styles of human public comments while presenting the entire body (one-shot learning). Each fake public comment generated by both ChatGPT and each public comment text written by a human were paired and had similar content. Next, we compared these texts from the perspective of their Japanese stylometric features. Especially, we analyzed no meaning stylometric features such as function words or sentence structures, rather than content words such as noun ‘cat’, verb ‘run’, and adjective ‘beautiful’, because the former features are not dependent on topic and genre of texts.

Thus, this study proposes the following hypothesis: Hypothesis 1: As shown in a previous study [5], the Japanese stylometric features of both GPT_zero_ texts (GPT3.5_zero_ and GPT4_zero_) are completely different from those of HM texts, even in public comments. Hypothesis 2: Both GPT_one_ texts (GPT3.5_one_ and GPT4_one_) are closer to the HM texts than the GPT_zero_ texts because of the effect of one-shot learning. Hypothesis 3: We can discriminate GPT-generated both types of fake public comments (both GPT_zero_ and GPT_one_) from human public comments using Japanese stylometric analysis, even if this study supposed hypothesis 2.

## Method

### Sample

As stated previously, we collected 100 Japanese public comments from the e-Gov website (https://www.e-gov.go.jp) published by Japanese national administrative agencies. There is no copyright problem because this website states that published information is not subject to copyright and can be freely used. Public comments covered various topics: telework security guidelines, eel aquaculture, support for the independence of the homeless, personal information protection law, etc. The number of characters in HM texts resulted in a mean of 661.3 (*SD* 132.0) and a median of 627.

Next, we make ChatGPT generated 100 texts (GPT-3.5) and 100 texts (GPT-4) in Japanese (i.e., GPT3.5_zero_ and GPT4_zero_ texts) with the next prompts: “You are ‘general citizen.’ Write a public comment (criticism, request, and opinion) about ‘title of the public comment.’.” If the attribute of the person who wrote the public comment was known to us, we change ‘general citizen’ to a specified attribute such as business person, lawyer, or doctor. The number of characters showed a mean of 604.3 (*SD* 61.3) and a median of 601.5 in GPT3.5_zero_ and a mean of 620.4 (*SD* 61.8) and a median of 621 in GPT4_zero_.

Lastly, as with GPT3.5_one_ and GPT4_one_ texts, we have ChatGPT generated two sets of 100 Japanese texts by having each ChatGPT (-3.5 and -4) emulate while presenting human public comments with the next prompts: “The following statement is a public comment (criticism, request, and opinion) submitted from a general citizen. Write a public comment similar in content and in writing style to this statement.” The number of characters of GPT3.5_one_ was a mean of 603.3 (*SD* 71.5) and a median of 594 and that of GPT4_one_ was a mean of 604.6 (*SD* 54.8) and a median of 621.

### Japanese stylometric features

We counted the frequency of occurrence of the next stylometric features and calculated the rate of frequency of occurrence within each text to avoid depending on the length of the count words of the texts.

#### Phrase patterns

Phrase patterns are regarded as effective features for authorship attribution in the Japanese language [7]. To analyze these features, we attached POS tags to each word using morphological analysis and divided the sentences into phrases using syntactic analysis. After the analysis, we focused on the combination of function words and POS of content words within each phrase: “noun + が (postpositional particle)”, “noun + noun + へ (postpositional particle) + の (postpositional particle)”, “実際 (adverb) + に (postpositional particle)”, and “noun + noun + noun + の (postpositional particle)” etc.

#### Parts-of-speech (POS) bi- and trigrams

The concept of *N*-gram is used in the field of quantitative linguistics to determine the frequency of a contiguous sequence of symbols (characters, words, phrases, etc.) in a sentence. Bigram is in the case of *N* = 2 (“preposition + noun” etc.), and trigram is in the case of *N* = 3 (“preposition + noun + adjective” etc.). Both POS trigrams and bigrams are effective stylometric features for authorship attribution [8].

#### Bigrams of postpositional particle words

The frequency of a contiguous sequence of postpositional particle words such as “を(case particle) + の (case particle)” and “は (binding particle) + が (case particle)” etc. A previous study on Japanese authorship attribution [9] reported effectiveness as a distinguishable feature but lower performance in AI detection tasks [5].

#### Positioning of commas

Positioning of commas is where the author used commas in sentences such as “は (binding particle) +,(comma),” “する (verb) +,(comma),” and “だ (auxiliary verb) +,(comma).” In other words, we focused on the words before the comma.

#### Function words

Preceding study of authorship attribution [10] and AI detection task [5] reported the function words as quite distinguishable features: “だ (auxiliary verb),” “また (conjunction),” and “は (postpositional particle).”

In Study 1, we confirmed which stylometric features were effective; in Study 2, we consolidated the effective features into integrated ones to examine incremental validity in verifying distinguishable performance levels.

In morphological analysis, we used the Japanese POS tagger Mecab [11] and attached POS tags (e.g., postpositional particle: “case particle,” “binding particle,” and “ending particle”). When syntactic analysis was conducted, we used the Japanese parser CaboCha [12].

### Analysis procedure

The current study essentially adopted the analysis procedure and statistical methods of Zaitsu & Jin [5] to compare the current results with prior results.

#### Study 1

To examine Hypotheses 1 and 2, we used classical multidimensional scaling (MDS). This statistical method can display the similarity between texts as distance; the more similar both texts are, the closer they are in dimensions. In MDS, the definitions of distances exist in various forms, and we used the symmetric Jensen-Shannon divergence distance (*d*_*SJSD*_) to compare 500 texts of five classes (HM, GPT3.5_zero_, GPT4_zero_, GPT3.5_one_, and GPT4_one_) in each Japanese stylometric feature because it is effective for authorship attribution [13] and AI detection [5]. The Eq (1) for the distance between x and y is shown below. We conducted MDS using the *cmdscale* function of the **stats** package of the R language.


$$d_{SJSD}{\left(x,y\right)}^{2}=\frac{1}{2}\Sigma_{i=1}^{n}\left(x_{i}\;\text{log}\frac{2x_{i}}{x_{i}+y_{i}}+y_{i}\;\text{log}\frac{2y_{i}}{x_{i}+y_{i}}\right)$$ (1)

#### Study 2

To verify the performance level for distinguishing among the three classes (GPT_zero_, GPT_one_, and HM), we used random forest (RF) and executed leave-one-out cross-validation (LOOCV). The RF classifier is a classical machine learning method similar to bagging. The reasons that we selected this classifier are follows: (1) The RF classifier is effective for authorship attribution [14] among several other classifiers and AI detection [5] in Japanese. (2) we investigate the effective stylometric features for distinguishing AI-generated texts from human-written ones. LOOCV is a type of cross-validation used to evaluate the generalization performance of a model. In this study, one text was excluded from the 500 texts as the testing set, and the RF classifier was trained using the remaining 499 texts to classify the testing text into one of three classes. These procedures were repeated 500 times using different test sets. We used the *randomForest* function of the **random Forest** package and set the number of decision trees to 1,000 and the other hyperparameters to default.

## Results

### Study 1: Comparison of text distributions of five classes (GPT3.5_zero_, GPT4_zero_, GPT3.5_one_, and GPT4_one_, HM)

**Fig 1:**
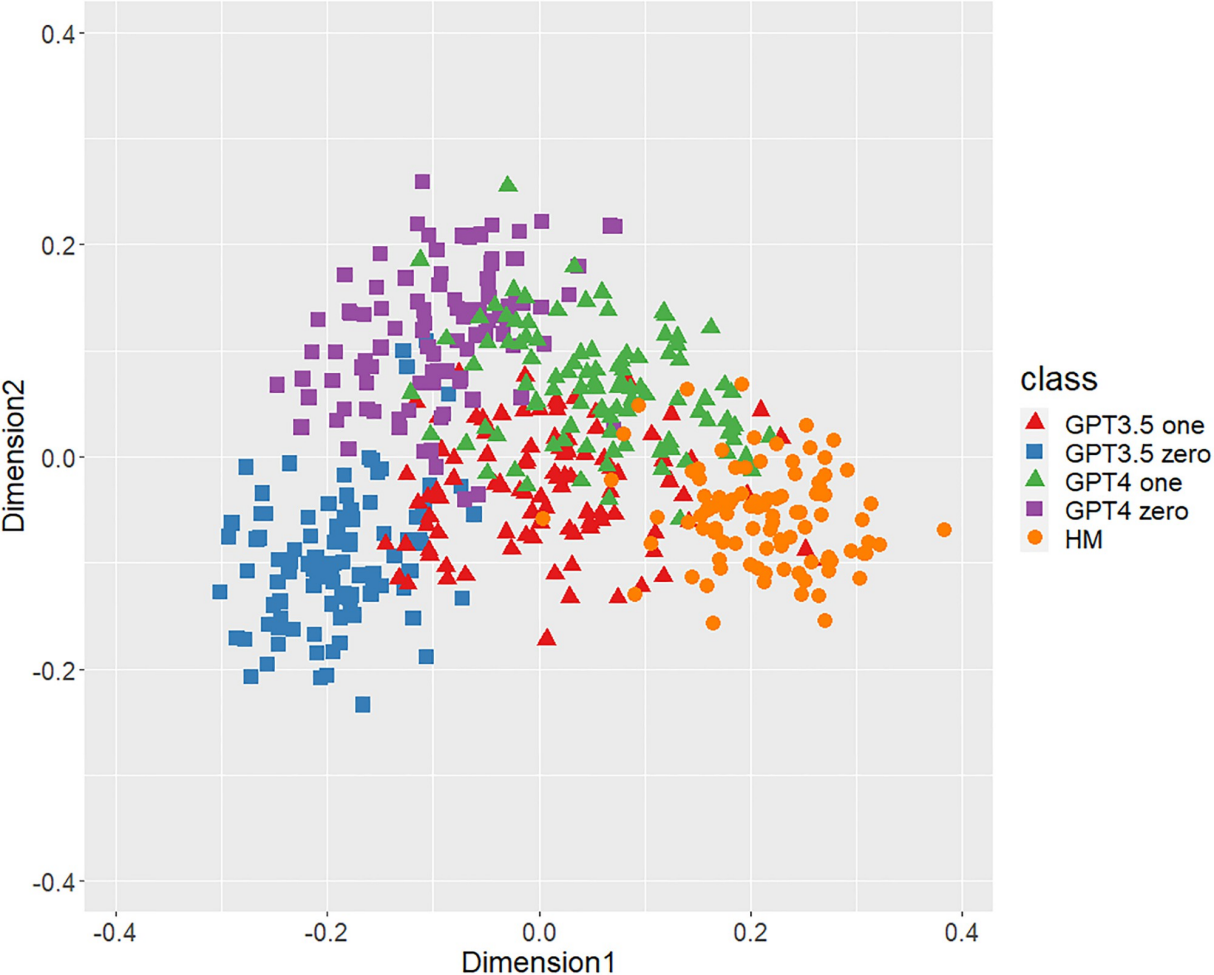
MDS configuration in five classes (GPT3.5_one_, GPT3.5_zero_, GPT4_one_, GPT4_zero_, and HM), focusing on the phrase patterns. “GPT3.5_one_” and “GPT4_one_” mean texts generated by GPT-3.5 and GPT-4 with one-shot learning. “GPT3.5_zero_” and “GPT4_zero_” indicate texts generated by GPT-3.5 and GPT-4 with zero-shot learning. “HM” means human-written public comment.

**Fig 2:**
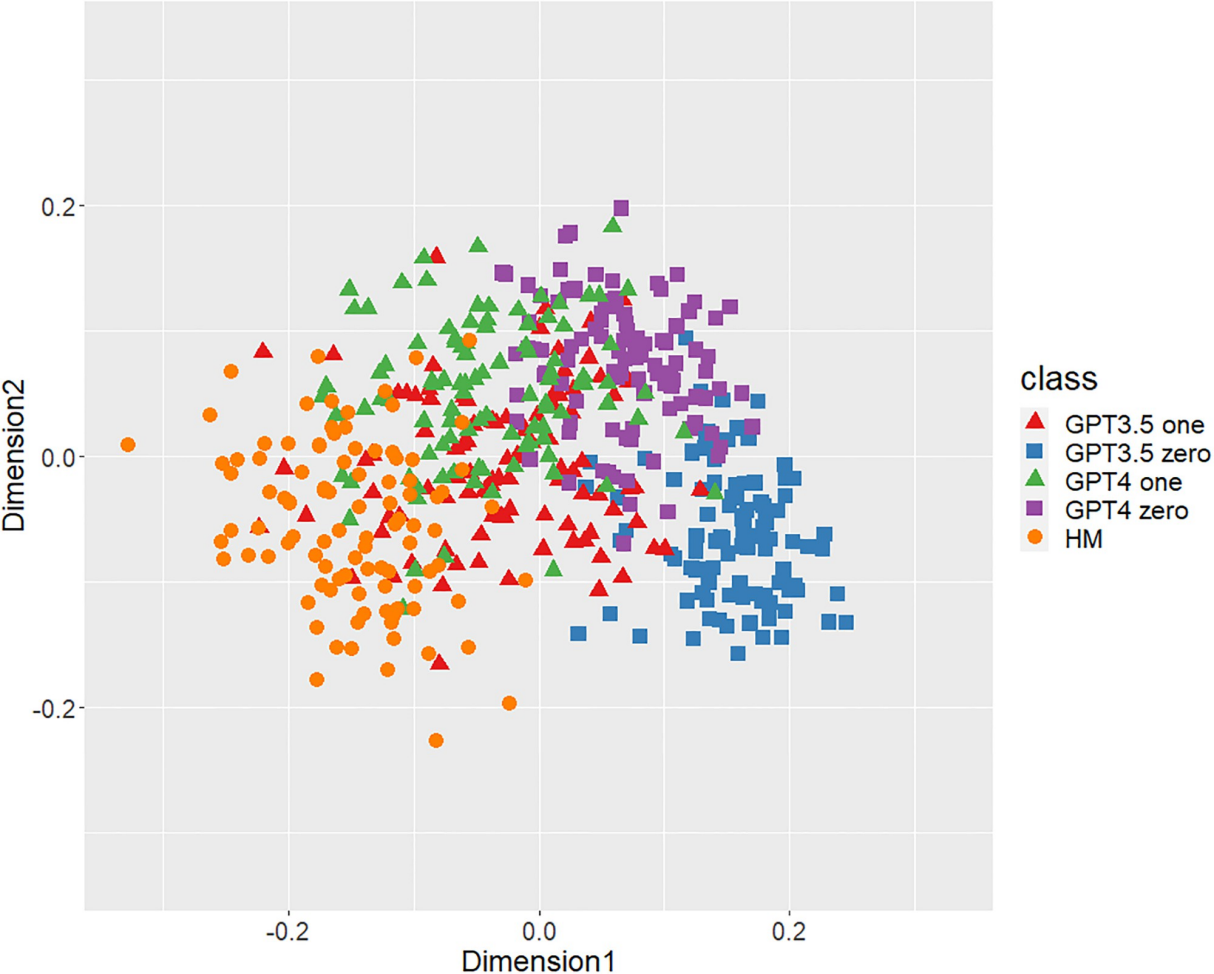
MDS configuration in five classes, focusing on the POS bigrams.

**Fig 3:**
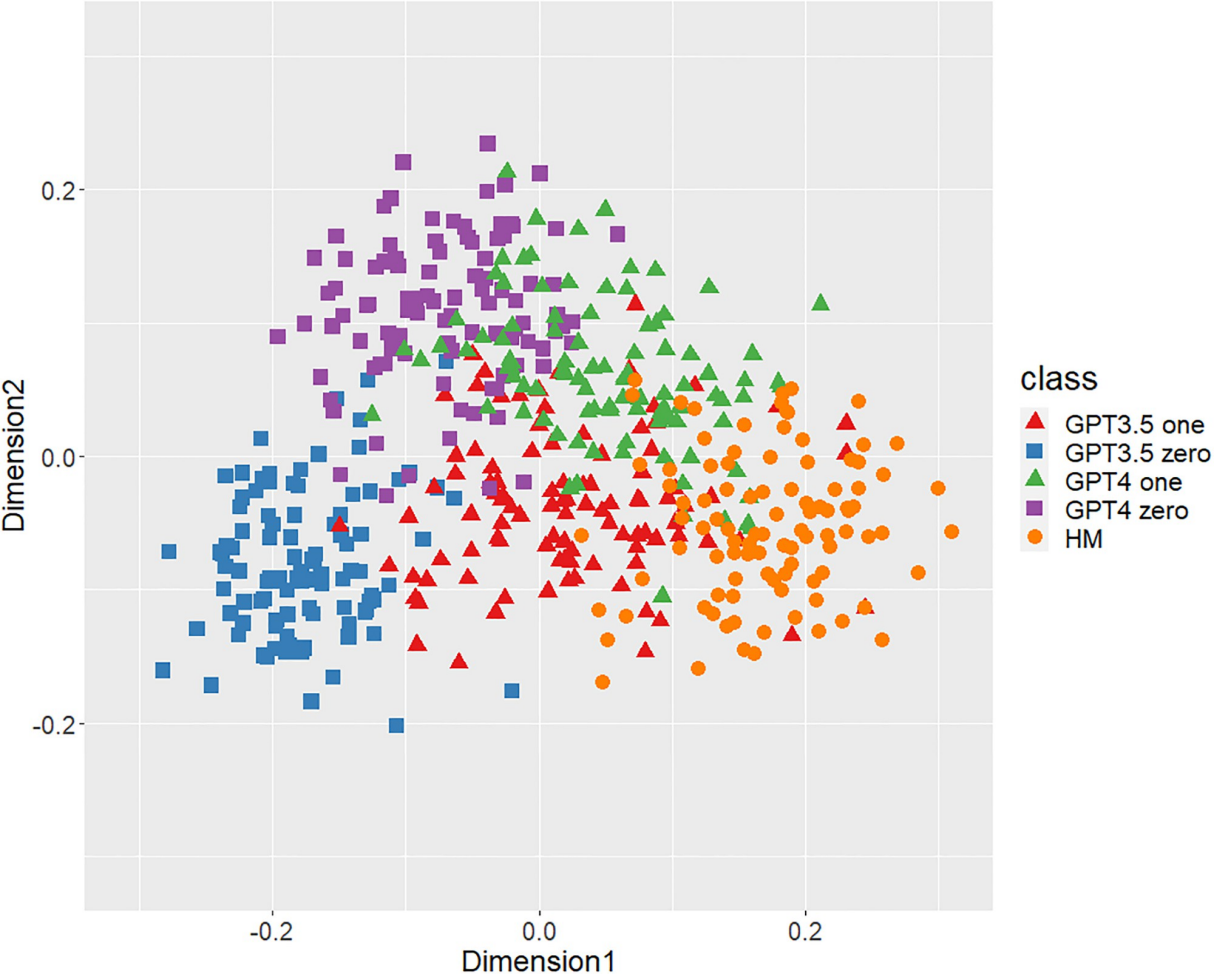
MDS configuration in five classes, focusing on the POS trigrams.

**Fig 4:**
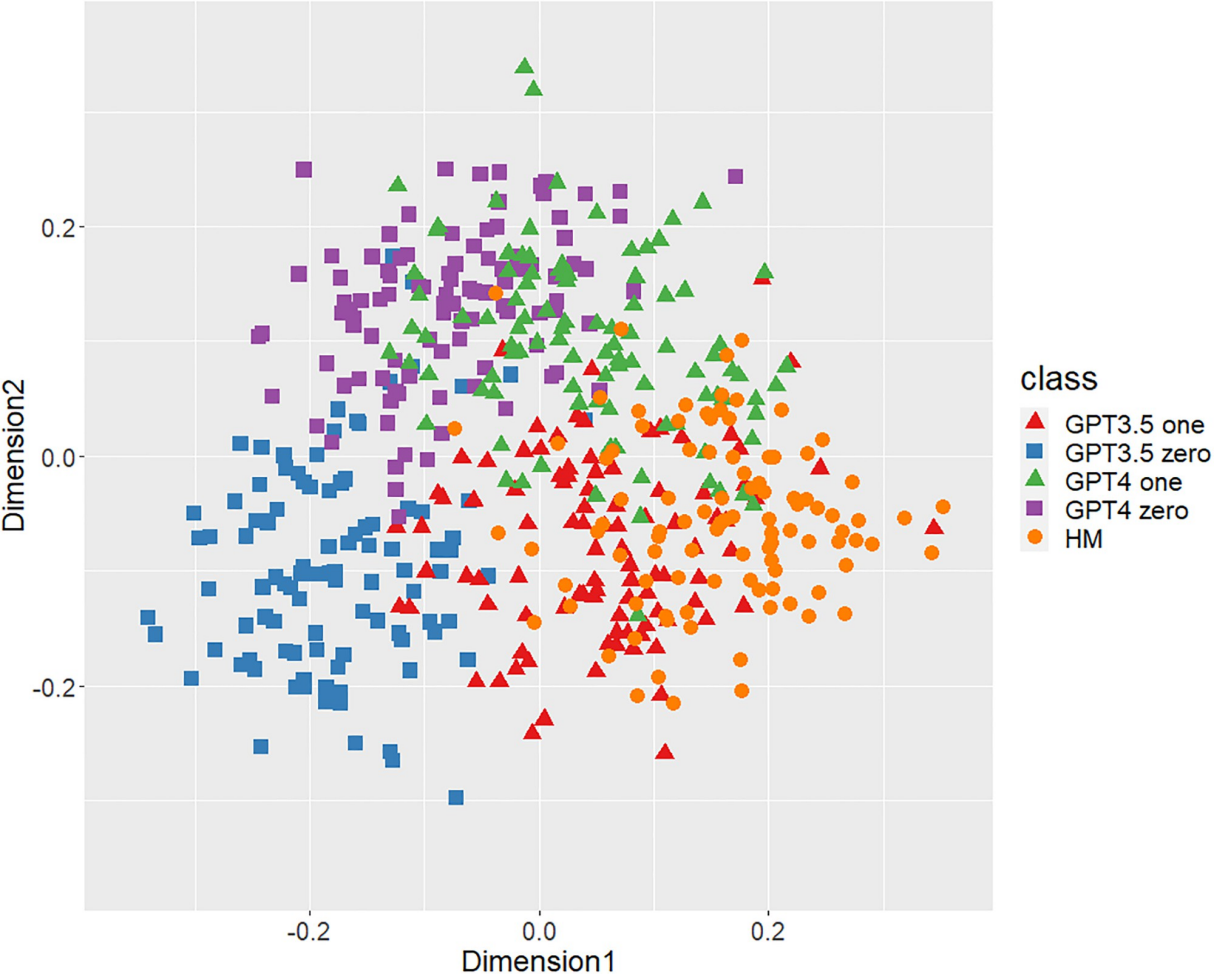
MDS configuration in five classes, focusing on the bigram of postpositional particle words.

**Fig 5:**
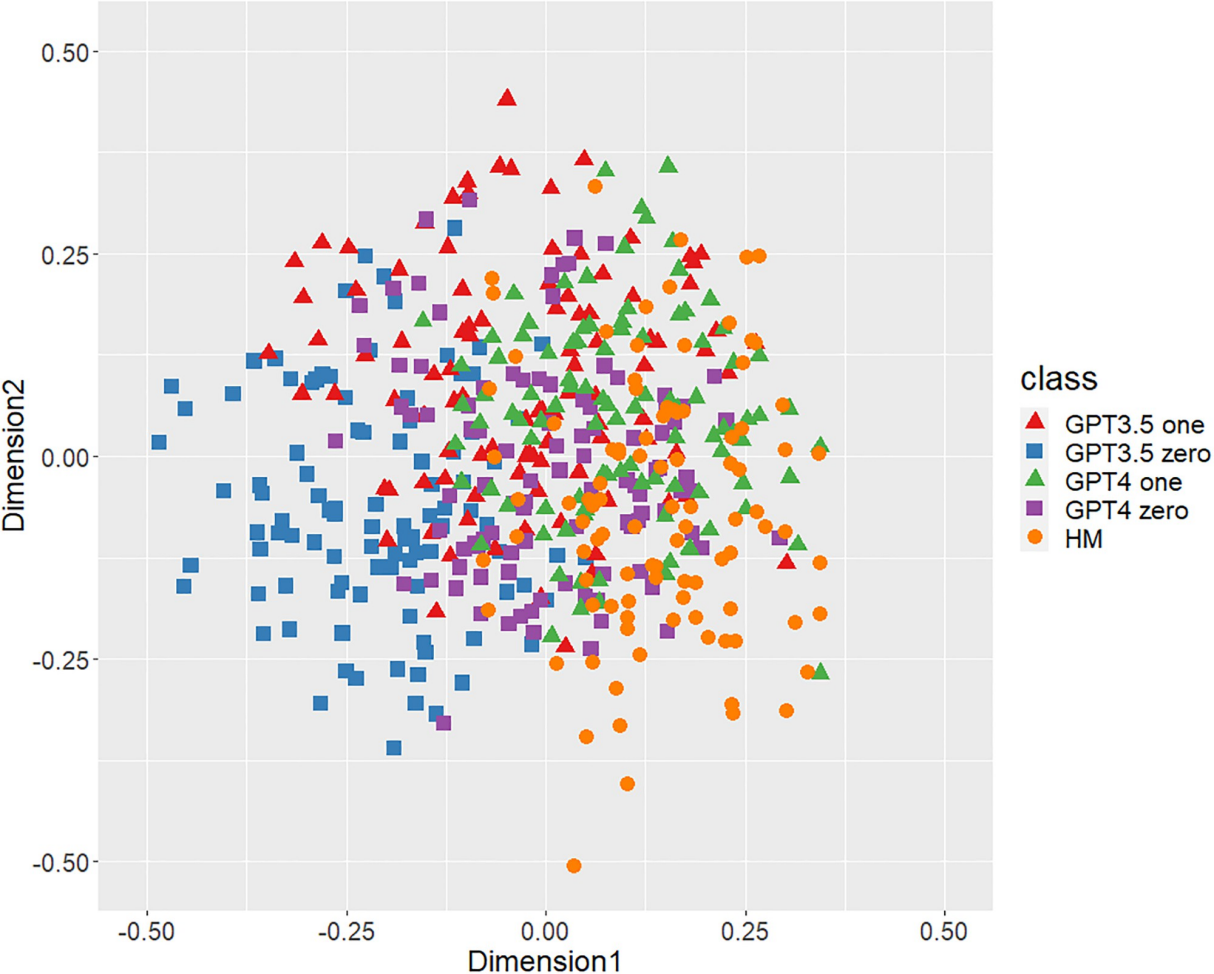
MDS configuration in five classes, focusing on the positioning of commas.

**Fig 6:**
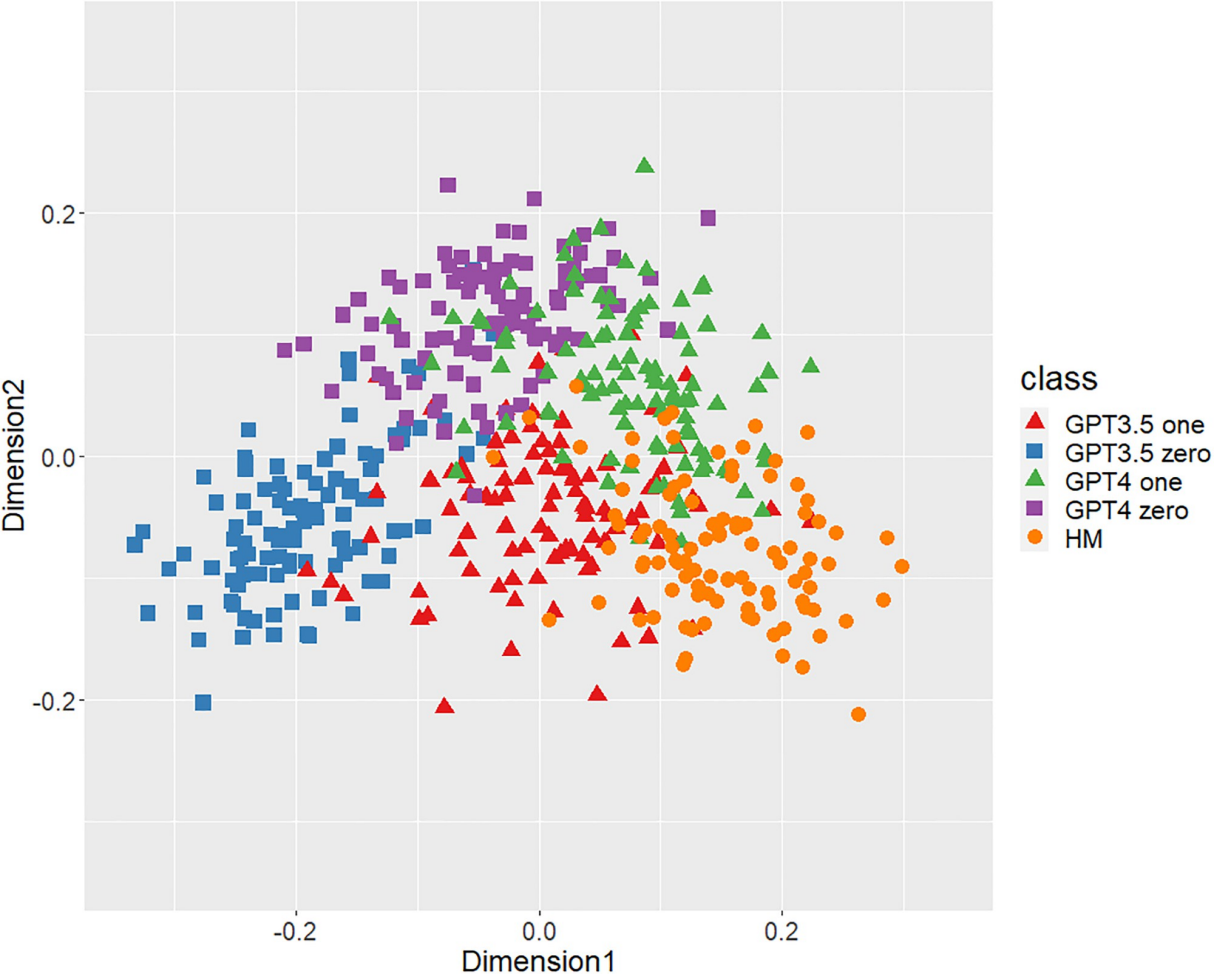
MDS configuration in five classes, focusing on the function words.

Figs 1–6 show the degrees of similarity and difference between the texts belonging to the five different classes separately for the six types of stylometric features. First, except for the positioning of commas in Fig 5, the stylometric features (Figs 1–4 and 6) appear to be HM texts that are completely separated from both GPT_zero_ texts. These results support hypothesis 1. Second, all but Fig 5 indicated that GPT3.5_zero_ and GPT4_zero_ have different distributions. Finally, according to all Figures except Fig 5, the distributions of both GPT3.5_one_ and GPT4_one_ are slightly closer to HM texts and are positioned between the distribution of GPT_zero_ texts and that of HM texts. Moreover, some GPT_one_ texts overlapped with HM texts.

Table 1 shows the means and standard deviations of the distances of the texts between GPT (GPT_zero_ and GPT_one_) and HM, corresponding to Figs 1–6. The distances between GPT_one_ and HM were shorter than those between GPT_zero_ and HM. This implies that GPT_one_ texts are more similar to human texts compared to GPT_zero_. These results support Hypothesis 2.

**Table 1:** The means and standard deviations of distances of the entire texts between GPT (GPT_zero_ and GPT_one_) and HM corresponding to each stylometric feature.

	GPT3.5_zero_ vs HM	GPT4_zero_ vs HM	GPT3.5_one_ vs HM	GPT4_one_ vs HM
Phrase patterns	0.82 (*SD* 0.04)	0.79 (*SD* 0.04)	0.77 (*SD* 0.05)	0.75 (*SD* 0.05)
POS bigrams	0.69 (*SD* 0.05)	0.68 (*SD* 0.05)	0.67 (*SD* 0.04)	0.68 (*SD* 0.04)
POS trigrams	0.94 (*SD* 0.03)	0.93 (*SD* 0.03)	0.92 (*SD* 0.04)	0.93 (*SD* 0.03)
Bigram of postpositional particle words	0.93 (*SD* 0.05)	0.91 (*SD* 0.05)	0.89 (*SD* 0.05)	0.89 (*SD* 0.05)
Positioning of commas	0.96 (*SD* 0.09)	0.94 (*SD* 0.09)	0.93 (*SD* 0.09)	0.92 (*SD* 0.09)
Function words	0.66 (*SD* 0.05)	0.63 (*SD* 0.05)	0.62 (*SD* 0.05)	0.61 (*SD* 0.05)

**Fig 7:**
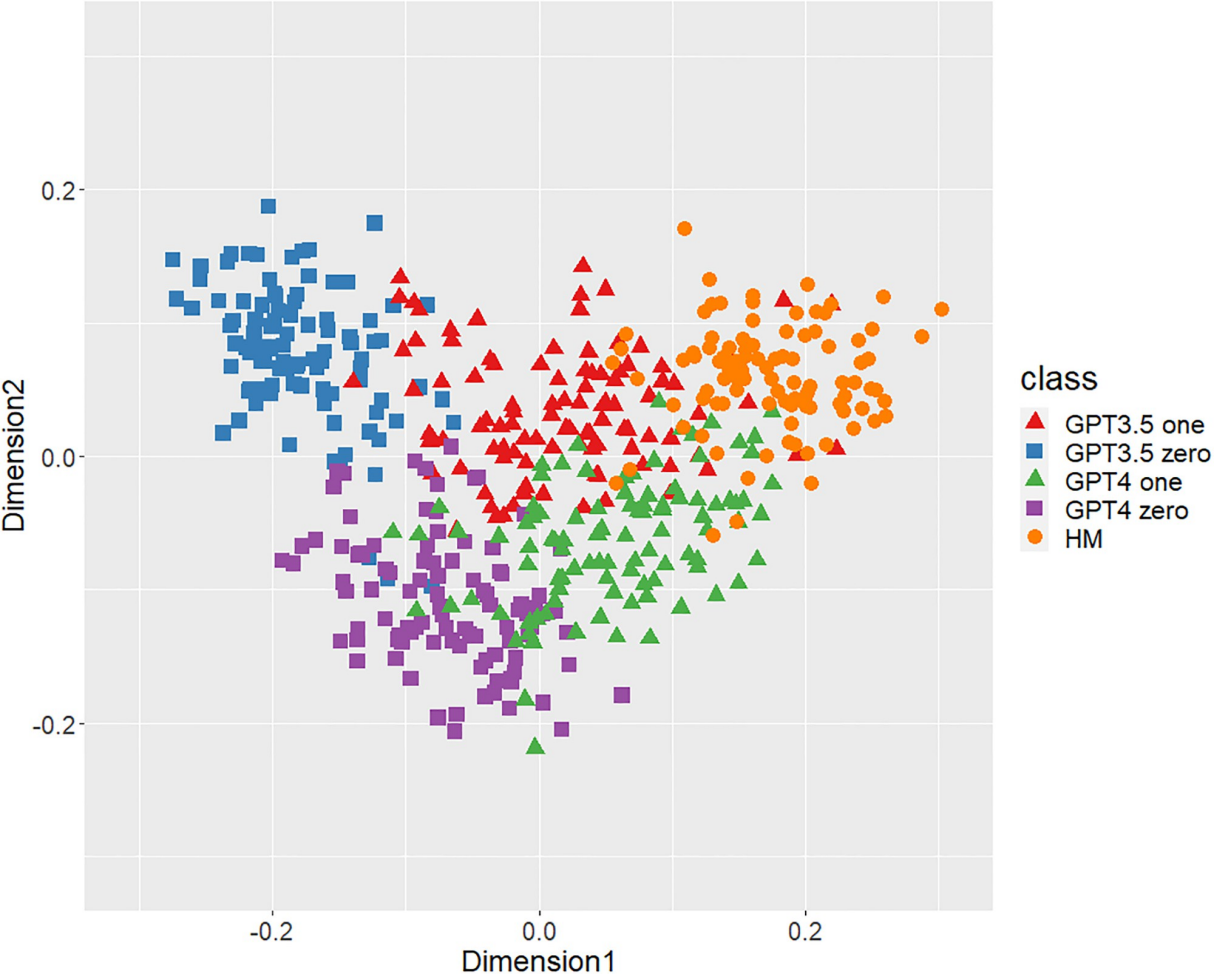
MDS configuration in five classes, focusing on integrated features (the phrase patterns, the POS bigrams and trigram, and the function words).

Only the positioning of commas (Fig 5) displays a mixture of all classes, which means that the positioning of commas is not an effective feature for classifying ChatGPT-generated and human-written public comments. Based on the above results, we judged phrase patterns (Fig 1), POS bigrams (Fig 2), POS trigrams (Fig 3), and function words (Fig 6) to be effective stylometric features for discriminating texts between ChatGPT and humans. Therefore, we integrated these four stylometric features and used them as “integrated features” for the next analysis. Fig 7 shows the MDS configuration of the texts, focusing on integrated features.

### Study 2: Evaluation of performance of RF classifier at LOOCV

First, we integrated GPT-3.5 and GPT-4 texts in each GPT-generated type, such as the three classes (GPT_zero_, GPT_one_, and HM). To evaluate the performance level for classifying the three classes using RF, we executed LOOCV and created confusion matrices for multiclass classification based on true classes and classified classes. Table 2 presents an example confusion matrix for these three classes. For instance, the cell of *a* in Table 2 means that RF classifier correctly judges text generated by ChatGPT with zero-shot learning as “GPT_zero_”, whereas the one of *c* indicates mistakes a judge as the text written by human. Next, based on the confusion matrix, the classification performance was assessed using the following metrics: “accuracy” in Eq (2), “recall” in Eqs (3A) to (3C), and “precision” in Eqs (4A) to (4C). The metric values were calculated for each class, together with the macro-average values (Eqs (5A) to (5B)).

$$Accuracy=\frac{a+e+i}{all\;texts\;\left(N=\;500\right)}$$ (2)

$$Recall\;for\;GPT_{zero}=\frac{a}{a+b+c}$$ (3A)

or

$$Recall\;for\;GPT_{one}=\frac{e}{d+e+f}$$ (3B)

or

$$Recall\;for\;HM=\frac{i}{g+h+i}$$ (3C)

$$Precision\;for\;GPT_{zero}=\frac{a}{a+d+g}$$ (4A)

or

$$Precision\;for\;GPT_{one}=\frac{e}{b+e+h}$$ (4B)

or

$$Precision\;for\;HM=\frac{i}{c+f+i}$$ (4C)

$$Macro\;average\;for\;recall=\frac{Recall\;for\;GPT_{zero}+GPT_{one}+HM}{3}$$ (5A)

$$Macro\;average\;for\;precision=\frac{Precision\;for\;GPT_{zero}+GPT_{one}+HM}{3}$$ (5B)

**Table 2:** Example of confusion matrix.

True class	Classified class		
	GPT_zero_	GPT_one_	HM
GPT_zero_	*a*	*b*	*c*
GPT_one_	*d*	*e*	*f*
HM	*g*	*h*	*i*

Additionally, we combined the class of GPT_zero_ and GPT_one_ texts as “GPT_zero and one_” and calculated “recall for GPT_zero and one_” and “precision for GPT_zero and one_”. Refer to the following Eqs (6A) and (6B) for details of the metric calculations. Among these performance metrics, we regard both “precision for HM” of Eq (4C) and “precision for GPT_zero and one_” of Eq (6B) as the most important performance metrics because our human want to accurately predict whether the sentences by an unknown author was written by ChatGPT or by a human.


$$Recall\;for\;GPT_{zero\;and\;one}=\frac{a+b+d+e}{a+b+c+d+e+f}$$ (6A)

$$Precision\;for\;GPT_{zero\;and\;one}=\frac{a+b+d+e}{a+b+d+e+g+h}$$ (6B)

Table 3 is a confusion matrix for the phrase patterns and each performance metrics are follows: accuracy (90.6%), recall for GPT_zero_ (95.0%), recall for GPT_one_ (83.5%), recall for HM (96.0%), recall for GPT_zero and one_ (97.5%), precision for GPT_zero_ (88.8%), precision for GPT_one_ (92.8%), precision for HM (90.6%), recall for GPT_zero and one_ (97.5%), precision for GPT_zero and one_ (99.0%), macro average for recall (91.5%), macro average for precision (90.7%). RF classifier can suggest which variables are effective for discrimination as “importance”. The importance indicated the following features are effective: “verb + れ + ます,” “noun + です,” and “noun + や.”

**Table 3:** Confusion matrix for the phrase patterns.

True class	Classified class		
	GPT_zero_	GPT_one_	HM
GPT_zero_	190	10	0
GPT_one_	23	167	10
HM	1	3	96

Table 4 shows the confusion matrix for the POS bigrams. The results of performance metrics are as follows: Accuracy (88.0%), recall for GPT_zero_ (97.5%), recall for GPT_one_ (76.0%), recall for HM (93.0%), precision for GPT_zero_ (87.1%), precision for GPT_one_ (92.7%), precision for HM (83.0%), recall for GPT_zero and one_ (95.3%), precision for GPT_zero and one_ (98.2%), macro average for recall (88.8%), macro average for precision (87.6%). According to the importance of RF, “auxiliary verb +. (period)” and “postpositional particle + noun” are regarded as effective features.

**Table 4:** Confusion matrix for the POS bigrams.

True class	Classified class		
	GPT_zero_	GPT_one_	HM
GPT_zero_	195	5	0
GPT_one_	29	152	19
HM	0	7	93

The results of performance metrics calculated from confusion matrix (Table 5) for the POS trigrams: Accuracy (87.2%), recall for GPT_zero_ (97.5%), recall for GPT_one_ (72.0%), recall for HM (97.0%), precision for GPT_zero_ (85.2%), precision for GPT_one_ (94.7%), precision for HM (81.5%), recall for GPT_zero and one_ (94.5%), precision for GPT_zero and one_ (99.2%), macro average for recall (88.8%), macro average for precision (87.1%). According to the importance of RF, “noun + auxiliary verb +. (period)” was regarded as an effective feature. Compared with the POS bigram, the performance level decreased slightly.

**Table 5:** Confusion matrix for the POS trigrams.

True class	Classified class		
	GPT_zero_	GPT_one_	HM
GPT_zero_	195	5	0
GPT_one_	34	144	22
HM	0	3	97

Table 6 presents the confusion matrix for the bigram of postpositional particle words. The performance levels are accuracy (76.0%), recall for GPT_zero_ (90.0%), recall for GPT_one_ (61.0%), recall for HM (78.0%), precision for GPT_zero_ (76.9%), precision for GPT_one_ (75.8%), precision for HM (74.3%), recall for GPT_zero and one_ (93.3%), precision for GPT_zero and one_ (94.4%), macro average for recall (76.3%), macro average for precision (75.7%). RF classifier indicated that “の + や”, “や + の”, and “や + を”are effective features.

**Table 6:** Confusion matrix for the bigram of postpositional particle words.

True class	Classified class		
	GPT_zero_	GPT_one_	HM
GPT_zero_	180	20	0
GPT_one_	51	122	27
HM	3	19	78

Table 7 shows the confusion matrix for the positioning of commas. The performance levels are lower as same as bigram of postpositional particle words: Accuracy (76.6%), recall for GPT_zero_ (88.0%), recall for GPT_one_ (68.5%), recall for HM (70.0%), precision for GPT_zero_ (78.9%), precision for GPT_one_ (77.8%), precision for HM (69.3%), recall for GPT_zero and one_ (92.3%), precision for GPT_zero and one_ (92.5%), macro average for recall (75.5%), macro average for precision (75.4%). Importance of RF indicated “する (verb) +, (comma)” and “において (postpositional particle) +, (comma)” as effective features.

**Table 7:** Confusion matrix for the positioning of commas.

True class	Classified class		
	GPT_zero_	GPT_one_	HM
GPT_zero_	176	18	6
GPT_one_	38	137	25
HM	9	21	70

The confusion matrix for the function words is displayed in Table 8. The performance levels were relatively higher: Accuracy (88.4%), recall for GPT_zero_ (95.0%), recall for GPT_one_ (78.5%), recall for HM (95.0%), precision for GPT_zero_ (86.0%), precision for GPT_one_ (91.3%), precision for HM (88.8%), recall for GPT_zero and one_ (97.0%), precision for GPT_zero and one_ (98.7%), macro average for recall (89.5%), macro average for precision (88.7%). The importance of RF indicated “や (postpositional particle)” and “です (auxiliary verb)” as effective features.

**Table 8:** Confusion matrix for the function words.

True class	Classified class		
	GPT_zero_	GPT_one_	HM
GPT_zero_	190	10	0
GPT_one_	31	157	12
HM	0	5	95

Finally, we integrated four effective features (the phrase patterns, the POS bigrams and trigrams, and the function words) and analyzed them using the integrated features. Table 9 presents the confusion matrix for the integrated features. The performances were slightly improved, compared to other features: Accuracy (91.6%), recall for GPT_zero_ (97.0%), recall for GPT_one_ (83.0%), recall for HM (98.0%), precision for GPT_zero_ (89.8%), precision for GPT_one_ (95.4%), precision for HM (89.1%), recall for GPT_zero and one_ (97.0%), precision for GPT_zero and one_ (99.5%), macro average for recall (92.7%), and macro average for precision (91.4%). This study demonstrated incremental validity because the integrated features achieved the best classification performance.

**Table 9:** Confusion matrixes for the integrated features (GPT_zero_ vs GPT_one_ vs HM).

True class	Classified class		
	GPT_zero_	GPT_one_	HM
GPT_zero_	194	6	0
GPT_one_	22	166	12
HM	0	2	98

For reference, the mean accuracies by 10-fold cross-validation showed next: (1) the phrase patterns: 86.0% (*SD* 3.9%), (2) the POS bigrams: 84.4% (*SD* 4.0%), (3) the POS trigrams: 82.4% (*SD* 4.7%), (4) the bigram of postpositional particle words: 75.4% (*SD* 6.7%), (5) the positioning of commas: 73.4% (*SD* 4.4%), (6) the function words: 83.2% (*SD* 3.8%), (7) the integrated features: 88.0% (*SD* 3.0%).

In addition to above the analyses, we calculated the classification performance metrics by focusing only on the integrated features to compare each GPT type to HM as follows: (1) GPT_zero_ (GPT 3.5_zero_ vs. GPT4_zero_) vs. HM and (2) GPT_one_ (GPT3.5_one_ vs. GPT4_one_) vs. HM. With regard to GPT_zero_ vs. HM, we can completely distinguish the GPT_zero_ texts from the HM (Table 10). Therefore, all performance metrics (accuracy, recall, and precision for GPT_zero_ vs. humans) resulted in 100%. However, in the case of GPT_one_ vs. human (Table 11), the classification performance slightly decreased compared to the other cases (GPT_zero_ vs. human) but maintained a high performance level: accuracy (95.3%), recall for GPT_one_ (94.5%), recall for HM (97.0%), precision for GPT_one_ (98.4%), and precision for HM (89.8%).

**Table 10:** Confusion matrixes for the integrated features (GPT 3.5_zero_ vs. GPT4_zero_ vs. HM).

True class	Classified class		
	GPT 3.5_zero_	GPT4_zero_	HM
GPT 3.5_zero_	98	2	0
GPT4_zero_	3	97	0
HM	0	0	100

**Table 11:** Confusion matrixes for the integrated features (GPT3.5_one_ vs GPT4_one_ vs HM).

True class	Classified class		
	GPT3.5_one_	GPT4_one_	HM
GPT3.5_one_	92	2	6
GPT4_one_	5	90	5
HM	3	0	97

## Discussion

This study examined whether we could distinguish between human public comments and ChatGPT-generated fake public comments (including ChatGPT-emulated humans) using Japanese stylometric analysis.

According to Study 1, the results of the MDS indicated that GPT_zero_ texts generated by presenting only the titles of public comments applicable to zero-shot learning were completely different from human-written texts. However, most of the GPT_one_ texts, which emulated human public comments (i.e., one-shot learning), were positioned between the distributions of GPT_zero_ and HM on the MDS dimension. Furthermore, some GPT_one_ texts overlapped slightly with the human texts. These results support Hypotheses 1 and 2: Japanese stylometric features of GPT_zero_ texts are completely different from those of human public comments, and GPT_one_ texts are more similar to human public comments than GPT_zero_. We consider that this center positioning of the GPT_one_ texts means not “closer from GPT_zero_ to human” but “closer from human to GPT_zero_” because GPT_one_ may start emulating and generating from human public comment. That is, GPT_one_ texts may be closer to GPT_zero_ texts by emulating and modifying the HM texts. Furthermore, according to the Figure (especially Figs 1–3), the texts of GPT4_one_ are farther away from the distribution of HM texts than GPT3.5_one_. These results suggest that the higher the performance of ChatGPT (i.e., GPT-4 at present), the easier it may be to distinguish emulated texts from human-written texts because higher-performance ChatGPT can more sophisticatedly rewrite human-written texts to make them closer to GPT_zero_ texts. Regardless of the lower word counts in the current study (appropriately 600 characters vs. 1,000 characters in a previous study [5]), the differences between the GPT with zero-shot learning and humans were larger in the current study than in the previous study. It is unclear why these results occurred because several factors, such as word count (600 words vs. 1,000 words) and categories (public comments vs. academic papers), were confounded. Fig 5 indicates that the positioning of commas had little distinguishable effect because almost all texts in each class overlapped. A previous study [5] demonstrated a certain effective level of comma positioning. We considered the possibility that the difference in genres (academic papers and public comments) influenced these results. Therefore, we need to further examine other genres of texts.

Study 2 showed that the best precision HM achieved was approximately 90% and that GPT_zero and one_ reached was 99.5%. Considering these results, it can be said that Hypothesis 3 was supported: we can discriminate fake public comments generated by ChatGPT from human public comments. Among the six Japanese stylometric features, phrase patterns indicated the best discriminable performance and POS bigrams and trigrams showed high classification accuracy. ChatGPT is not good at rewriting texts taking these features into consideration because these stylometric features (the phrase patterns, POS bigrams, and POS trigrams) are regarded as a deeper structural aspect of sentences. However, the present study revealed low performance of the positioning of commas, particularly in the GPT emulation. ChatGPT can easily rewrite this feature in sentences because of linguistically low-level features. While presenting human public comments and making ChatGPT emulate, we confirmed ChatGPT often just paraphrased words (e.g., from “ignorant” to “fool”). Therefore, presently, even if we analyze other languages, we may be able to distinguish sentences between generative AI and humans by focusing on deeper structures.

Above the results of this study limited Japanese language. Zaitsu & Jin [5] also pointed out that Japanese language have different notation formats (Kanji, Hiragana, and Katakana) and no space between words as opposed to English. Therefore, we need conduct similar verification for other languages as well. In addition, we need collect and analyze larger sample size of human-written and AI-generated public comments for the purpose of generalization of this study.

Recently, the disinformation generated by AI, such as fake news, has become a problem worldwide because these fakes are instantly and widely generated. Disinformation has certainly caused chaos in the human world; therefore, we need techniques to control generative AI, including sophisticated classifiers.

## Conclusion

The current study concluded that (1) the stylometric features of Japanese public comments were completely different from ChatGPT-generated texts by presenting only the titles of public comments (i.e., zero-shot learning). (2) The public comments generated by the one-shot trained ChatGPT with human-generated public comments are more similar to human public comments than the public comments from the zero-shot trained ChatGPT. (3) Although limited to this study sample (Japanese language, approximately 600 characters, and learning method of ChatGPT), at present, we can discriminate ChatGPT-generated fake public comments from human public comments through stylometric analysis.

## Supporting information

S1 Data (CSV)

S2 Data (CSV)

S3 Data (CSV)

S4 Data (CSV)

S5 Data (CSV)

S6 Data (CSV)

## References

[1] OpenAI [Internet]. Introducing ChatGPT; c2022 [cited 2023 May 31]. https://openai.com/blog/chatgpt.

[2] Köbis N, Mossink LD. Artificial intelligence versus Maya Angelou: Experimental evidence that people cannot differentiate AI-generated from human-written poetry. Comput. Hum. Behav. 2021;114: 106553.

[3] Clark E, August T, Serrano S, Haduong N, Gururangan S, Smith NA. All that’s ‘Human’ is not gold: Evaluating human evaluation of generated text. Proceedings of the 59th Annual Meeting of the Association for Computational Linguistics and the 11th International Joint Conference on Natural Language Processing (ACL-IJCNLP-2021), Virtual Conference. 2021;1: 7282–7296.

[4] Desaire H, Chua AE, Isom M, Jarosova R, Hua D. Distinguishing academic science writing from humans or ChatGPT with over 99% accuracy using off-the-shelf machine learning tools. Cell Rep. Phys. Sci. 2023;4(6): 101426. doi: 10.1016/j.xcrp.2023.101426 37426542 PMC10328544

[5] Zaitsu W, Jin M. Distinguishing ChatGPT(-3.5, -4)-generated and human-written papers through Japanese stylometric analysis. PLOS ONE. 2023;18(8): e0288453. doi: 10.1371/journal.pone.0288453 37556434 PMC10411719

[6] Brown T, Mann B, Ryder N, Subbiah M, Kaplan J, Dhariwal P, et al. Language models are few-shot learners. Adv. in Neural Inf. Process. Syst. 2020;33, 1877–1901.

[7] Jin M. Bunsetsu patan ni motoduita bunshou no kakite no shikibetsu [Authorship identification based on phrase patterns]. Jpn. J. Behaviormetrics. 2013;40(1), 17–28.

[8] Jin M. Hinshi no marukohu sen i no joho wo motiita kakite no dotei. Proceedings of the 32th Annual Meeting Behaviourmetric Society, Tokyo, Japan. 2004;384–385.

[9] Jin M. Joshi no N-gram modelu ni motoduita kakite no shikibetsu [Authorship attribution based on N-gram models in postpositional particle of Japanese]. Jpn. J. Math. Ling. 2002;23(5), 225–240.

[10] Zaitsu W, Jin M. Tekisuto mainingu niyoru hisshashikibetsu no seikakusei narabini hantei tetsuduki no hyoujunka [Accuracy and standardized judgment procedures for author identification]. Jpn. J. Behaviormetrics. 2018;45(1):39–47.

[11] Kudo T, Yamamoto K, Matsumoto Y. Applying conditional random fields to Japanese morphological analysis. Proceedings of the 2004 Conference on Empirical Methods in Natural Language Processing (EMNLP-2004), Barcelona, Spain. 2004; 230–237.

[12] Kudo T, Matsumoto Y. Japanese dependency analysis using cascaded chunking. Proceedings of the 6^th^ Conference on Natural Language Learning (CoNLL-2002), Taipei, Taiwan. 2002; 63–69.

[13] Jin M, Jiang M. Text clustering on authorship attribution based on features of punctuation usage in Chinese. Inf. 2013;16(7):4983–4990.

[14] Jin M, Murakami M. Randamu foresutohou ni yoru bunshou no kakite no doutei [Authorship identification using Random Forests]. Proc. Inst. Stat. Math. 2007;55(2):255–268.

