# Supplementary material for: Can we spot fake public comments generated by ChatGPT(-3.5, -4)?: Japanese stylometric analysis expose emulation created by one-shot learning
Source: PLoS One. 2024 Mar 13;19(3):e0299031. doi: 10.1371/journal.pone.0299031 (PMC10936841; doi:10.1371/journal.pone.0299031)
Supplement: Attachment — Submitted filename: Response to Reviewers.docx [file pone.0299031.s007.docx]

Answer and Response to Editor and Reviewer 1 comments:

Thank you for your constructive review.

I revised several points along with your comments.

Please confirm revised paper.

Thank you.

1. Additional explain of contribution of this study in the Introduction.

We additionally explained contribution of this study (p4, l80).

2. equations number and citation

We give number in all equations and appropriately cited.

3. “Further each variable used in the equation needs to be discussed properly.”

Sorry. We don’t understand this comment. Would you explain this more.

4. The training and testing ratio

We conducted LOOCV. Among 500 sample, we used 499 sample for training and 1 sample for test. In this study, we did not set validation sample because we fixed hyperparameters.

5. About Random Forest Algorithm

We politely explained why we used random forest (p8, l192).

6. The limitation of this study.

We added the limitation of this study in discussion, along with reviewer’s comment (p20, l406).

7. emoji and sarcasm

There was no emoji in our public comments. Usually, “emoji” are used in informal circumstances, for example, exchange between acquaintances. On the other hand, public comments are formal circumstances.

Furthermore, we did not confirm“sarcasm”. Moreover, we focused on non-content words, so this stylometric analysis is not affected by sarcasm.

　8. Capstion in the figures

We revised and added caption explain.

Answer and Response to Reviewer 2 comments:

Thank you for your constructive review.

I revised several points along with your comments.

Please confirm revised paper.

Thank you.

1.The limitation of this study.

We added the limitation of this study in discussion, along with reviewer’s comment (p20, l406).

2.Revised references

We revised references, especially added details about conferences, along with reviewer’s comment.
