# Supplementary material for: Can we spot fake public comments generated by ChatGPT(-3.5, -4)?: Japanese stylometric analysis expose emulation created by one-shot learning
Source: PLoS One. 2024 Mar 13;19(3):e0299031. doi: 10.1371/journal.pone.0299031 (PMC10936841; doi:10.1371/journal.pone.0299031)
Supplement: Attachment — Submitted filename: Response to Reviewers.docx [file pone.0299031.s008.docx]

Answer and Response to Editor and Reviewer 1 comments:

Thank you for your constructive review.

I revised several points along with your comments.

Please confirm revised paper.

Thank you.

1. Out of 500 sample , 499 is taken for training and 1 is taken for testing. Standard traing and testing ratio is 80-20, 70-30. In the current mauscript case the model will go in the overfitting condtion, so k-fold cross validation is required to check the overfitting condtion.

LOOCV (Leave-one-out cross-validation), conducted in this study, is generally less prone to overfitting compared to other cross-validation methods like k-fold cross-validation, especially when the dataset is small like this current study. Therefore, we used this method.

But for reference, we conducted 10-fold cross validation, and wrote these results.

2. Each variable/parameters used in equation need to be disccused. Example in equation 6A, (parameters a,b,c,d,e,f) need to be discussd. What is a, what is b , like wise.

Along with this comments, we additionally explain in details (p16, l377).

3.Change part of the title

Before this submission, the title is ““Can we spot fake public comments generated by ChatGPT(-3.5, -4)?: Japanese stylometric analysis expose emulation **through** one-shot learning”. But I think “through one-shot” may lead to misunderstanding for readers that not “expose emulation created by one-shot learning” but “expose by one-shot learning”. So we want to change part of the title to “Can we spot fake public comments generated by ChatGPT(-3.5, -4)?: Japanese stylometric analysis expose emulation **created by** one-shot learning”
